# Supplementary material for: Mortality predictors of hospitalized patients with COVID-19: Retrospective cohort study from Nur-Sultan, Kazakhstan
Source: PLoS One. 2021 Dec 22;16(12):e0261272. doi: 10.1371/journal.pone.0261272 (PMC8694457; doi:10.1371/journal.pone.0261272)
Supplement: Attachment — Submitted filename: Response to Reviewers PLOS One2.docx [file pone.0261272.s003.docx]

## 25 November 2021,

## Nur-Sultan, Kazakhstan

## Re: PLOS ONE: PONE-D-21-25018

# Title: Mortality predictors of hospitalized patients with COVID-19: Retrospective Cohort Study from Nur-Sultan, Kazakhstan

**To: Academic Editor**

**Muhammad Tarek Abdel Ghafar, M.D**

**PLOS ONE**

**Dear Dr. Muhammad Tarek Abdel Ghafar**

We would like to express our deepest gratitude for the time and efforts of the Reviewers and the Editor spent on our manuscript. All the outstanding comments enlightened us, and they will ultimately improve the scientific and practical content of our paper.

We are resubmitting the revised version that incorporates the suggestions made by the Reviewers and feel that the manuscript is greatly enhanced as a result. Enclosed below you will also find the point-by-point reply to all comments.

We hope that our manuscript in its current revised format is now suitable for publication in PLOS ONE

We look forward to hearing your decision.

Sincerely yours,

Dr. Aliya Sailybaeva

**CORRESPONDING AUTHOR**

**Answers to the review Comments to the Author**

- *Please review your reference list to ensure that it is complete and correct. If you have cited papers that have been retracted, please include the rationale for doing so in the manuscript text, or remove these references and replace them with relevant current references. Any changes to the reference list should be mentioned in the rebuttal letter that accompanies your revised manuscript. If you need to cite a retracted article, indicate the article’s retracted status in the References list and also include a citation and full reference for the retraction notice*.

Answer: The retracted article from [Giovanni Ponti, Monia Maccaferri, Cristel Ruini, Aldo Tomasi, Tomris Ozben. Biomarkers associated with COVID-19 disease progression. Crit Rev Clin Lab Sci. 2020 : 1–11. Published online 2020 Jun 5. doi: 10.1080/10408363.2020.1770685] is included with original citations and the Reference list is filled by references from retracted article.

Based on previous studies according to Giovanni et all [24], which is described the work Qin et al. [22] we analyzed markers, in particular, white blood cells and CRP in a cohort of 450 patients with confirmed COVID-19, and found that the severe course of the disease is characterized by an increased level of white blood cells and an increase in C-reactive protein, compared with patients with a moderate course. Similarly, in other works, for example, in Henry et al. It was also concluded in a meta-analysis of 21 studies involving 3377 patients with confirmed COVID19 that patients with a severe course of the disease and fatal outcomes had very high levels of white blood cells, compared with patients with a mild course of the disease and recovered patients. [23, 24] What we observed in our patients as well.

**References**

22.	Qin C, Zhou L, Hu Z, et al. Dysregulation of immune response in patients with COVID-19 in Wuhan, China. Clin Infect Dis. 2020. DOI:10.1093/cid/ciaa248

23.	Henry BM, de Oliveira MHS, Benoit S, et al. Hematologic, biochemical and immune biomarker abnormalities associated with severe illness and mortality in coronavirus disease 2019 (COVID-19): a meta-analysis. Clin Chem Lab Med. 2020.

24.	Giovanni Ponti, Monia Maccaferri, Cristel Ruini, Aldo Tomasi, Tomris Ozben. Biomarkers associated with COVID-19 disease progression. Crit Rev Clin Lab Sci. 2020 : 1–11. Published online 2020 Jun 5. doi: 10.1080/10408363.2020.1770685

-The bar graphs with error are replaced with the Box plot with whisker.
